# Translational models of 3-D organoids and cancer stem cells in gastric cancer research

**DOI:** 10.1186/s13287-021-02521-4

**Published:** 2021-09-06

**Authors:** Kenly Wuputra, Chia-Chen Ku, Kohsuke Kato, Deng-Chyang Wu, Shigeo Saito, Kazunari K. Yokoyama

**Affiliations:** 1 Graduate Institute of Medicine, Kaohsiung Medical University, Kaohsiung, 80708 Taiwan; 2 Regenerative Medicine and Cell Therapy Research Center, Kaohsiung Medical University, Kaohsiung, 80708 Taiwan; 3 Cell Therapy and Research Center, Kaohsiung Medical University Hospital, Kaohsiung, 80756 Taiwan; 4 Department of Infection Biology, Graduate School of Comprehensive Human Sciences, The University of Tsukuba, Tsukuba, 305-8577 Japan; 5 Department of Gastroenterology, Department of Internal Medicines, Kaohsiung Medical University Hospital, Kaohsiung, 80756 Taiwan; 6 Waseda Research Institute of Science and Engineering, Waseda University, Tokyo, 169-0051 Japan; 7 Saito Laboratory of Cell Technology, Yaita, Tochigi 329-1571 Japan

**Keywords:** Cancer microenvironment, Cancer stem cells, Human gastric organoids, Gastric cancer, Translational research, Tumor suppressor genes, Patient-specific organoid library

## Abstract

It is postulated as a general concept of cancer stem cells (CSCs) that they can produce cancer cells overtly and repopulate cancer progenitor cells indefinitely. The CSC niche is part of a specialized cancer microenvironment that is important to keep the phenotypes of CSCs. Stem cell- and induced pluripotent stem cell (iPSC)-derived organoids with genetic manipulation are beneficial to the investigation of the regulation of the microenvironment of CSCs. It would be useful to assess the efficiency of the cancer microenvironment on initiation and progression of cancers. To identify CSCs in cancer tissues, normal cell organoids and gastric cancer organoids from the cancerous areas, as well as iPSCs, were established several years ago. However, many questions remain about the extent to which these cultures recapitulate the development of the gastrointestinal tract and the mechanism of *Helicobacter pylori*-induced cancer progression. To clarify the fidelity of human organoid models, we have noted several key issues for the cultivation of, and differences between, normal and cancerous organoids. We developed precise culture conditions for gastric organoids in vitro to improve the accuracy of the generation of organoid models for therapeutic and medical applications. In addition, the current knowledge on gastrointestinal CSC research, including the topic of CSC markers, cancer cell reprogramming, and application to target cancer cell plasticity through niches, should be reinforced. We discuss the progression of cancers derived from human gastric organoids and the identification of CSCs.

## Introduction

The human stomach is divided into two major regions, i.e., the corpus/fundus and the antrum [1]. The epithelium layer is formed into the fundus gland, consisting of endocrine cells, tuft cells, stem cells, parietal cells (with acid-secretion ability), mucus-producing cells, and chief cells (with pepsinogen-secretion ability) in the corpus [2]. The antrum gland contains endocrine cells, mucus cells included gastrin-producing cells, and tuft cells, but comprises chief cells. The leucine-rich repeat-containing receptor (LGR5)^+^ stem cells generate to most cell types in the antrum. The human fundic and antral glands are also organized into three basic regions: the base area, medial isthmus, and surface pit area. Undifferentiated stem cells produce the isthmus. Chief, endocrine, and parietal cells are present in the base area of fundic glands, whereas endocrine, LGR5^+^ stem cells, the parietal and chief cells, are found in the base of antral glands. Tuft cells also reside on both glands. The stomach is mainly composed of these gastric epithelial and mesenchymal tissues. Thus, the understanding of the functional development of the gastric epithelium and mesenchymal tissues is critical to elucidate the composition of the stomach.

**Fig. 1:**
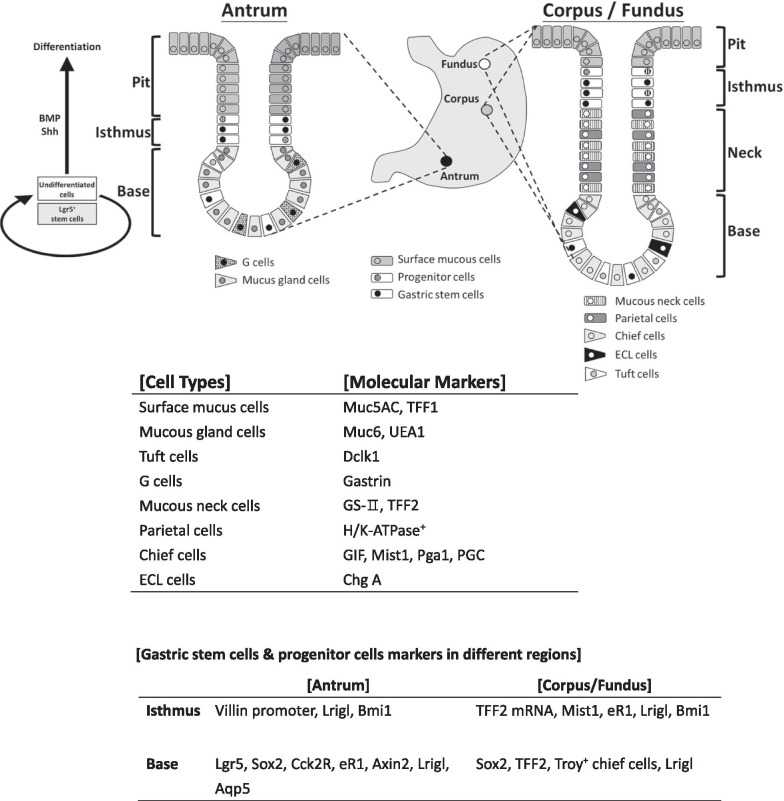
Gastric stem cells in human antrum and corpus/fundic area. In the antrum, Notch signaling affects the balance between proliferation and differentiation in the human stem cells. Both gastrin and acetylcholine are niche factors. In corpus/fundic area, Shh and BMPs are restricted to the area of isthmus and neck of fundic gland to affect LGR5^+^ stem cells for differentiation. Stromal cells (CXCL12^+^ endothelial- and CXR4^+^ innate lymphoid-cells) contribute to the corpus/fundic stem cells niches partially through production of Wnt5a. EGF, FGF10 and WNT play critical roles in control of self-renewal and pluripotency of gastric stem cells in the base gland of antrum and the isthmus of corpus/fundus [3, 5]. Each cell type and molecular markers are listed. Muc5AC, mucin 5AC; TFF1, trefoil factor 1; GS-II, Griffonia simplicifolia II; TFF2,trefoil factor 2; Muc6, mucin 6; UEA1; Ulex europaeus agglutinin 1; GIF, gastric intrinsic factor Pga1, pepsinogen 1; PGC, pepsinogen C; ChgA, chromogranin A; Dclk1, double cortin-like kinase 1

**Fig. 2:**
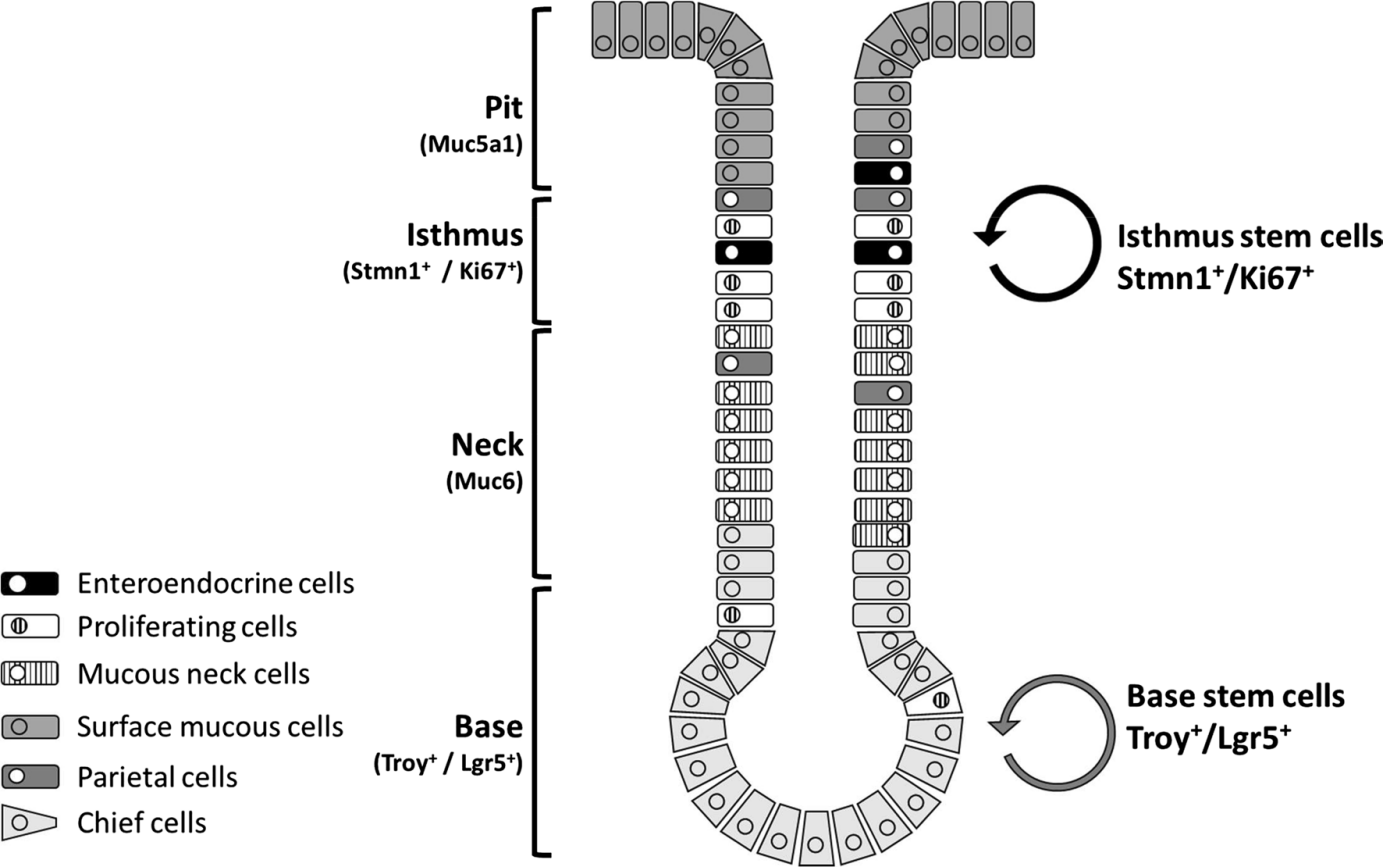
Schematic representation of mouse gastric corpus gland. In mouse gastric corpus, two different stem cell population such as isthmus stem cells (IsthSCs) and base stem cells included Troy^+^ and Lgr5^+^ cells are maintained [4]. IsthSCs are multi-potent and cycling actively and maintaining the pit-isthmus-neck regions through a process of stochastic self-renewal. IsthSCs are localized between pit and neck and are characterized as expressions of Stmn1 and Ki 67. As IsthSCs move to displace upward or downward from the Isthmus region and become sub-lineage restricted with co-expression of lower Muc5aca and Muc6 before terminal differentiation into the respective cell types, the gland base is maintained by TROY^+^ or LGR5^+^ (Chief) stem cells, which are mostly quiescent

Recent studies have suggested that different populations of gastric stem cells exist in different parts of the stomach and develop into distinct stem cell populations during stomach development. Cells expressing the most prominent stem cell markers, such as LGR5 and cholecystokinin 2 receptor (CCK2R^+^ [Numb^+^, Dll1^high^]), axin 2 (AXIN2), and aquaporin 5 (AQP5), are present in the antrum region. Cells expressing trefoil factor 2 (*TFF2*) mRNA, and muscle TWIST1 (MIST1) cells and mature chief cells expressing tumor necrosis factor receptor superfamily member 19 (TROY) are present in the corpus region. Furthermore, cells with SOX2, Runx enhancer 1 (eR1), leucine-rich repeats and immunoglobulin-like domains protein 1 (LRIG1), and B cell-specific Moloney murine leukemia virus integration site 1 (BMI1) are present in both the antrum and corpus [3]. The gastric corpus stem cells comprise the following two different stem cell populations: isthmus and base stem cells in the base region (TROY^+^ or LGR5^+^ cells) [4] (Figs. 1 and 2, Table 1). The epithelia of gastric corpus glands in mice are formed by two different stem cells: the proliferating stem cell population in the isthmus area (characterized by Stathmin 1 [STMN 1] and Ki67) and the quiescent population in the base area characterized by specific expression of TROY or LGR5 (Fig. 2) [4]. The stomach stem cells are localized in three different regions. The marker molecules of these stem cells have been reported elsewhere (Fig. 1) [3].

**Table 1:** Comparison of gastric stem cells

	Antrum (Human) [Ref. 3, 5]	Isthmus (Mouse) [Ref. 4]	Corpus/Fundus (Human) [Ref. 3, 5]
Location of cells	Isthmus, Gland base	Isthmus	Isthmus, Gland base, Chief cells
Differentiation	Expand bidirectionally for isthmus	At gland base stem cells migrate upward from the basal zone	Migrate into two independent zones; Slow-cycling stem cells in the base and active-cycling cells to the pit-isthmus-neck region
Markers	(Isthmus) Lrig1, Bmi1 (Gland base) LGR5, SOX2, CCK2R, CR1, AXIN2, AQP5	(Isthmus) Rapid cycling isthmus stem cells: Stmn1, Ki67 (Neck) weak Muc6 (Pit) weak Muc5ac (Base) Troy or Lgr5	(Isthmus) TFF2mRNA,MIST1, eR1, LRIG1, BMI1 (Gland base) SOX2, TFF2, LRIG1, TROY^+^-Chief cells
Offspring	Surface mucous cells, Mucous gland cells, G cells, Tuft cells	Rapid cycling Isthmus Stem cells -Muc5ac, Muc6	Surface mucous cells, Mucous neck cells, Parietal cells, ECL cells
Niche factors	Wnt, Notch, Gastrin, Ach, EGF, FGF10	A process of punctuated neutral drift dynamics	WNT, BMPs, SHH, EGF, FGF10

Human antrum and corpus/fundus stem cells were characterized by the location, markers and differentiation potency through niche factors as described in ref. 3. Mouse isthmus stem cells were reported recently to commit the rapid cycling stem cells expressing Stmn1 and Ki67 [4]

Gastric organoids from fundic, isthmus, and antral origins contain the glandular epitheliums and the mesenchyme layer [5]. The epithelium includes all types of mucus cells, endocrine cells, and chief cells. The fundic organoids include parietal cells expressed proton-pump and can release acid by histamine-response. However, human gastric organoids (HGOs) are not completely equal to adult stomach tissue. For example, HGOs seem to be fetal, i.e., the mesenchyme is not composed in smooth muscle in vitro. Moreover, they do not include vasculature or an enteric nervous system (ENS) [6]. These problems will be solved in the latter part.

Stem-cell-related pluripotency genes, such as *OCT4*, *SOX2*, *KLF4*, *MYC*, *LIN28*, and *NANOG*, are expressed in various types of cancer cells. For instance, *OCT4*, *SOX2*, and *NANOG* (OSN) are upregulated in bladder, breast, colorectal, prostate, and renal carcinogenic cells under the conditions of cultivation of embryonic stem (ES) cells and iPSCs [7]. Pluripotency-inducing transcriptional pathways were found in the aggressive cancers and showed strong resistance to clinical treatments, leading to poor survival. The similarities between cancer formation (tumorigenesis or carcinogenesis) and the iPSC-inducing reprogramming process are suggested to be driven by overlapping molecular signaling pathways [8]. The risk of tumorigenesis is happened by some mutations of oncogenes and/or tumor suppressor genes during the transformation of stem cells into cancers with niche cells [9].

### Candidate genes related to human gastric cancer

Recently, RNA sequencing of human gastric adenocarcinoma (GAC) and human gastric adenoma yielded The Cancer Genome Atlas database (http://www.gdc.cancer.gov), and the gastric cancer mRNA expression-based stemness index (mRNAsi) was also reported [10–12] (Table 2). mRNAsi was enhanced in gastric cancer tissues compared with normal gastric tissues (*P* < 0.0001). The following 16 genes were identified as the key genes in gastric cancer: budding uninhibited by benzimidazoles 1 (*BUB1*), budding uninhibited by benzimidazoles 1 homolog beta (*BUB1B*), non-SMC condensin I complex subunit H (*NCAPH*), kinesin family member 14 (*KIF14*), Rac GTPase-activating protein 1 (*RACGAP1*), DNA repair and recombination protein RAD54-like (*RAD54L*), TPX2 microtubule nucleation factor (*TPX2*), *KIF15*, *KIF18B*, centromere protein F (*CENPF*), dual specificity protein kinase (*TTK*), *KIF4A*, shugoshin-like (*SGOL*), Polo-like kinase 4 (*PLK4*), X-ray repair cross complementing 2 (*XRCC2*), and chromosome 1 open reading frame 12 (*C1orf12*). These genes are related to the spindle cell components, sister chromatid segregation, motor activity, cell-cycle, and homologous recombination. The prognosis of patients with gastric cancer is related to the expression of *RAD54L*, *TPX2*, and *XRCC2* genes [10]. The protein‒protein interaction network and the Cane Genome Atlas database showed that the expression of fibronectin 1 (FN1), serpin family E member 1 (SERPINE1), secreted protein acidic and rich in cysteine (SPARC) are related to the poor prognosis of GACs [11]. Identification of these target genes aids in understanding the mechanism of gastric cancer development and enables drug developments to prevent gastric cancer.

**Table 2:** Human gastric cancer-related genes

Genes	Names	Characteristics	References
BUB1	Budding is not inhibited by benzimidazoles	S/T kinase, Mitosis, Spindle checkpoint	[10]
BUB1B	Budding is not inhibited by benzimidazole homolog beta	kinase in spindle checkpoint, cell cycle, mitosis	[10]
NCAPH	Non-SMC condensin 1 complex subunit H	Condensin complex, Mitotic chromosome	[10]
KIF14	Kinesin family member 14	Kinesin superfamily, Microtubule motor proteins	[10]
KIF15	Kinesin family member 15	Kinesin superfamily, Microtubule motor protein	[10]
KIF18B	Kinesin family member 18B	Kinesin superfamily, Microtubule motor protein	[10]
KIF4A	Kinesin family member 4A	Kinesin superfamily, Microtubule motor proteins	[10]
RACGAP1	Rac GTPase activating protein 1	Component of centralspindlin complex, Cytokinesis, Cell growth & differentiation	[10]
RAD-54L	DNA repair and recombination protein RAD54-like	DEAD-like helicase superfamily, Repair of DNA double strand breaks	[10]
TPX2	TPX Microtubule nucleation factor	Cell cycle, Mitotic control of PLK1, G_2_/M Transition	[10]
CENPF	Centrosome protein F	Associated with centrosome, Kinetochore complex, Chromosome segregation	[10]
TTK	Dual specificity protein kinase	Mitotic check point, Accurate segregation of chromosome	[10]
SGO	Shugoshin	Protection centromeric cohesion from cleavage during mitotic prophase	[10]
PLK4	Polo family of S/T protein kinase 4	Control centriole duplication	[10]
XRCC2	X-ray repair cross complementing 2	A member of Rec A/Rad51 related protein family, Chromosome stability and repair DNA damage, DNA double strand break	[10]
ClorfI2	Egl-9 family hypoxia inducible factor 1	4-hydroxyprolin in HIFα protein, Cellular oxygen sensor	[10]
FN1	Fibronectin	Cell adhesion, Motility, Opsonization, Would healing, Cell shape	[11]
SERPINE1	Serpin family member 1	Serine protease inhibitor superfamily, Inhibitor of tPA and urokinase inhibitor of fibrinolysis	[11]
SPARC	Secreted protein acidic acid cysteine rich	Cysteine-rich acidic matrix-associated protein	[11]
MLLs	Myeloid/Lymphoid or Mixed-lineage Leukemia protein member	Methyltransferase of histone 3K4	[12]
ARD1A	AT-rich interactive domain-containing protein 1A	SWI/SNF family, Helicase/ATPase. Chromatin remodeling	[12]
EZH2	Enhancer of zeste homolog 2	Histone-lysine-N-methyltransferase enzyme, Chromatin remodeling	[12]
PCAF	p300/CBP-associated factor	Lysine acetyltransferase 2B (KAT2B), Transcriptional coactivator associated with p53	[12]
UTX (KDM6A)	Lysine -specific demethylase 6A	Demethylation of Lysine residue of histone, specifically H3K27	[12]

Human gastric adenocarcinoma (GAC) and human gastric adenoma (GAS) were examined by the RNA sequencing and gastric cancer mRNA expression-based stemness index (mRNAsi) [see refs. 10–12]. The gastric cancer related genes are listed. mRNAsi was significantly upregulated in gastric cancer tissues compared to normal gastric tissues (*P* < 0.0001)

### Epigenetics alteration of gastric cancer

The epigenetic driver genes such as myeloid/lymphoid or mixed-lineage leukemia (*MLL*)s, AT-rich interaction domain 1A (*ARID1A*) and enhancer of zeste 2 polycomb repressive complex 2 subunit (*EZH2*), P300/CBP-associated factor (*PCAF*), and lysine demethylase 6A (*KDM6A*; *UTX*), and their downstream targets are frequently mutated and affected in GACs [12].

Cancer progression is caused by phenomena of epigenesis, such as tumorigenic-enhancer reactivation in both normal cells and cancer cells. CSC-targeted anticancer therapies for the purpose of inhibiting epigenetic modifiers are promising. For example, inhibitors of DNA methyltransferase (DNMT), histone deacetylase (HDAC), bromodomain, and extraterminal motif proteins have been permitted by the United States Food and Drug Administration and they are going to therapeutic trials for various malignant cancers [13]. The chromatin regulatory complexes of polycomb repressive group (PcG)/trithorax group (TrxG) can control the reprogramming of cancer cells, and they perform a critical role in the progression of CSCs [8]. A chromatin modifier, the linker histone variant H1.0, can control the cell division and promote the cancer cell differentiations. Therefore, H1.0 expression must have an obstructive property against tumorigenesis in vivo [14]. It has been demonstrated that the ARID1A, which is a subunit of the ATP-dependent chromatin remodeling complex switch/sucrose non-fermentable (SWI/SNF), functions to suppress the tumorigenesis of colon cancers, and its molecular loss enables the activation of the tumorigenic program for the formation of colon adenocarcinoma in mouse models [15].

### Tumor suppressor genes and pluripotency-inducing factors

*TP53* is a tumor suppressor gene which is involved in cell cycle arrest for DNA repair. In addition, it inhibits the mutation of genes during cell division and plays a critical role in apoptosis [16]. Inhibition of such tumor suppressor genes, i.e., *P53* or the phosphatase and tensin homolog gene (PTEN), increased the efficiency of reprogramming to iPSCs [17]. Conversely, cyclin D inhibits to reprogram somatic cells to generate stem cells [18].

The expression of pluripotency-inducing genes, such as OCT4, SOX2, KLF4, an c-MYC (OKSM), is led by iPSCs, induces dedifferentiation in the body, and produces pluripotent cells in various bodies, teratomas, and dysplasias [19]. One of the reprogramming factors, c-MYC, is well known to overexpress in many cancers and inhibits differentiation, and promotes tumor formation in the absence of p53. As described earlier, OKSM, as well as *NANOG* and *LIN 28*, is protooncogenes, and stem cell-like cells inherently have carcinogenic potential. However, in colon and gastric carcinoma cells, *KLF4* has p21^Cip1^-dependent tumor suppression activity, thus inhibiting tumor progression and carcinogenesis [20].

The development of metastasis in CSCs is caused by their chemoresistance capacity, which is related to the expression of *OCT4* and *NANOG* [21]. Therefore, targeting *OCT4* or *NANOG* should be a rational strategy for therapeutic application in certain types of cancers. Short hairpin RNA (shRNA)-mediated *NANOG* knockdown in human gastric cancer cells decreased the characteristics of malignant cancer cells by increasing apoptosis and arresting cell cycle at the S phase [22]. Reexpression of stemness marker genes, such as *c-MYC*, which is a WNT target, in the murine intestine caused tumor initiation together with dedifferentiation and triggered stem cell-like properties in gastric cells [19]. This tumorigenesis initiation relied on the activation level of Wnt and developed exclusively on augmentation of Wnt. Thus, a single signal pathway might be sufficient to commit the reprogramming of somatic cells to tissue-specific CSCs. These findings appear to prove the potential of the inhibition of stem cell marker genes together with synergistic chemotherapy for arresting tumorigenesis. Nevertheless, molecular, epigenetic, and cellular events in reprogramming of cancer cells do not appear to be this simple; rather, they are so complex.

### Cancer stem cells

Cancer stem cells (CSCs) have been suggested to elucidate their properties of self-renewal properties via clonal cancer progression and clonal long-term repopulation [23]. CSCs have also been identified in glioblastoma and breast carcinomas [24, 25] since the presence of CSCs was firstly identified in acute myeloid leukemia [26]. In contrast to other cancer cells, CSCs can generate cancer cells overtly and repopulate progenitor cells indefinitely. The intratumor heterogeneity may be due to the various grades of differentiation ability between the CSCs and their progeny. Moreover, CSCs comprise less than 5% of cell populations which express a CD44 and respond to the epithelial surface antigen [27]. The combination of CD44 and CD24 was identified as an effective gastric CSC marker [28]. CD133 was first used to isolate CSCs from colon carcinoma [28]. Subsequently, another study showed that CD133 is not restricted to CSCs [3, 29]. The combination of epithelial cell adhesion molecule (EpCAM) with CD44 or CD54 with CD44 was also used for the isolation of gastric CSCs [29]. Moreover, in gastrointestinal cancers, CSCs are mostly positive for LGR5, because LGR5^+^ cells could produce gastric cancers [30]. A cancer treatment strategy involving extermination of most CSC populations using antibody–drug conjugates (ADCs) against specific CSC surface markers has been established. Studies using ADCs have excluded LGR5^+^ CSCs, and ADCs have shown an anticancer potential in mouse intestinal cancer models [30].

**Fig. 3:**
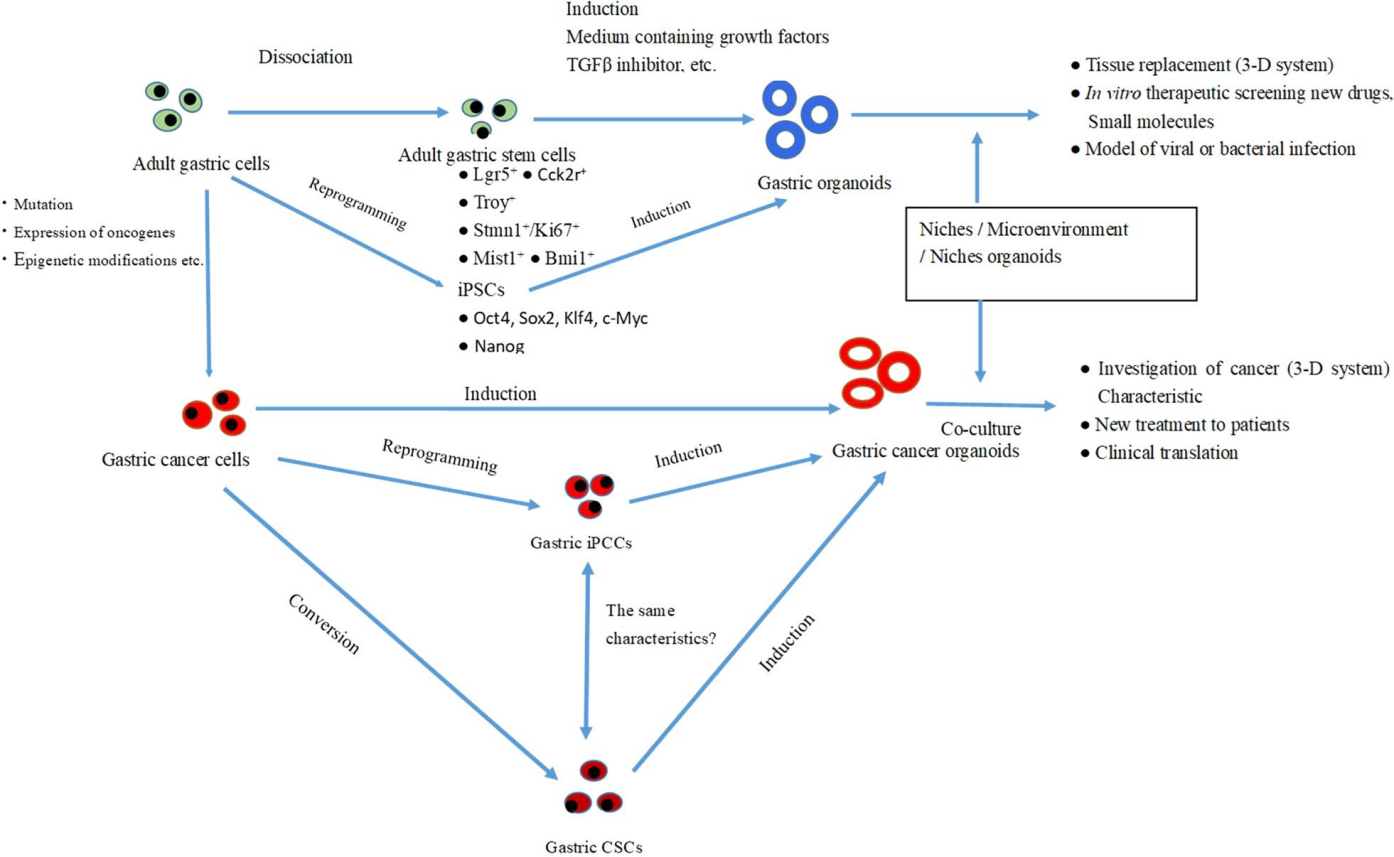
Schematic representation of generation of gastric normal and cancer organoids from gastric normal stem cells and cancer stem cells. The schematic models of the generation of gastric organoids and cancer organoids from respective cancer stem cells. The application of the organoids was for the regeneration of each tissue and screening of the new drugs, microbiota infection, and virus infection as well as the stomach cancers

Genetic and single-cell RNA sequencing studies showed that the heterogeneity of gastric cancer cells can be explained using the following two models: (1) the clonal evolution model and (2) a combination of the hierarchical and stochastic theories models [31]. The clonal evolution model suggests that driver mutations contribute to the expansion of cancer clonal cells by conferring a fundamental growth advantages to these cells. The recurrent genetic changes alter the phenotypes and functional characteristics of gastric cancer cells, which enables them to adapt to their cancer cells to their environments, suggesting a critical role of clonal evolution in gastric cancer to generate the heterogeneity and development. The combination model proposes adaptation of the cancer cells to their environment by changes in their gene expression patterns and generation of various subclones. Primitive CSCs have the natures of unlimited self-renewal and multilineage differentiation capacity. This results in the hierarchical structure of tumors. These CSCs evolve into different subclones and then sometime to evade the immunological system. Furthermore, the dominant clones produce their respective hierarchical cancer cells in the tumor areas, while other remaining cells are removed. These CSCs from models (i) or (ii) are required for the cancer microenvironments, such as the niches, to maintain the properties and plasticity of CSCs. Thus, as described above, studying the interaction between CSCs and their niches is critical for understanding the progression of the gastric cancer cells [31]. Moreover, the epigenetic regulation and signaling pathways also affect the development of CSCs and their niches. Here, we introduce an approach using three-dimensional (3-D) organoids to understand the interaction between gastric cancer CSCs and niches. These organ-level interactions will be investigated further to understand how the gastric cancer develops and progress, and how the gastric CSCs and the microenvironments interact to commit the tumor program. The use of gastric SCs and gastric CSCs and their organoids are useful for study of regeneration and the therapeutics application as well as cancer prevention (Fig. 3).

### Tumorigenic or tumor-suppressive potential of reprogrammed pluripotent gastric cancer cells

Induced reprogramming of malignant (chronic myeloid leukemia) cells with abnormal or deleted p53 raised the efficiency of the generation of iPSCs and the frequency of tumor formation after the transplantation of these reprogrammed malignant cells into animals. Reprogrammed iPSCs acquired insensitivity to treatment with the kinase inhibitor imatinib [32]. In the strict sense, it may be better to prescribe these cancer cell-derived iPSCs as induced pluripotent cancer stem cells (iPCSCs) or induced pluripotent cancer cells (iPCCs) to distinguish the normal iPSCs derived from noncancerous, healthy cells. Immediately after the successful establishment of iPSCs from human somatic cells [9], this reprogramming method was applied to several types of cancer cells to examine alterations in the tumorigenesis of iPCCs [33]. Miyoshi et al. performed to reprogram human gastrointestinal cancer cell lines, and eight iPCC (or iPCSC) lines were established from 20 cell lines by overexpressing OKSM, *LIN28*, and *NANOG* with the addition of shRNAs for tumor suppressor genes [33]. The resultant iPCCs were less tumorigenic compared with their parental cells. Several articles have reported the lower success rate in the generation of iPCCs among similar types of cancer cell lines [34]. Moreover, the lower efficiencies of cancer cell reprogramming are another problematic research subject. These lower efficiencies might be caused by various intricate mechanisms that are involved in the induction of cancer cell reprogramming [33, 34].

As a model for investigating carcinogenesis, the application of cancer cell reprogramming must be worthwhile regarding its operation. A few reports have demonstrated the repression of tumorigenicity in reprogrammed iPCCs derived from cancer cells, including gastrointestinal cancer [33, 35]. In our study that used *OCT4* and *JDP2* as pluripotency-inducing reprogramming factors, the iPCCs generated from a gastric cancer cell line inhibited the tumorigenic capacity of xenografts in SCID mice compared with that of parental CS12 cells by switching off the expression of the bone morphogenic protein 7 (BMP7) [36]. A growing body of experimental findings revealed that the high-level expression of BMP7 was correlated with its oncogenic function of increasing tumor invasion and metastasis, which led to the proposal that the reprogramming of gastric cancer cells might be a good option for the development of cancer research, with a possible impact on future therapies and the prevention of cancer initiation. In contrast, we generated iPCCs from a DAOY medulloblastoma cell lines by introduction of the same combination of *OCT4* and *JDP2* as reprogramming inducers; these iPCCs exhibited a higher tumorigenic potential in xenografts than did the parental DAOY cells, and the generation of a CSC-like state via forced expression of *JDP2* was noted in that experiment. This difference in tumorigenic capacity between cancer cell lines may be linked to the heterogeneity of plasticity and/or epigenetics. Noncoding RNA alterations, chromatin alterations, and histone modifications near the mutated DNA regions are also master key events in the conversion of tumorigenic characteristics, which are often controlled by special DNA elements named super-enhancers [6]. Thus, the protocols used for reprogramming cancer cells must be optimized according to the respective types of cancer.

### Generation of fundic organoids

The generation of HGOs in vitro is essential for the similar manipulation of growth factor signaling to that in vivo. To produce foregut tissue (spheroids) from human pluripotent stem cell (hPSC)-derived definitive endoderm, Noggin inhibits BMP to induce the enhanced expression of SOX2 and repressed expression of CDX2 [6]. The changes from 2-dimensional (2-D) endoderm into 3D-gut spheroids are performed by the activation of Wnt and FGF4 [6]. To promote antral identity with PDX1 expression, the 3-D spheroids require to inhibit BMP furthermore and to activate retinoic acid (RA) pathway, together with enhancing epidermal growth factor (EGF) signaling. Thus, this cascade is essential for the development of antral-specific organoids.

The protocol of formation of fundic organoids was more complexed because the signals that commit to the formation of the fundus were not known. The canonical Wnt signaling is essential to identify fundic specification. The pro-fundic role of Wnt signaling is distinct from its developmental role of repressing the anterior endoderm the early stage, thus exemplifying the concept that the function of signals has different roles at diverse developmental stages. Continuous activation of WNT is sufficient for promotion of a fundic epithelial fate by inhibiting PDX1 in posterior foregut cultures and results in the generation of fundic organoids expressing markers of mucus and chief cells. But it is not enough to promote the differentiation of parietal cells, which is needed as an additional process to inhibit MEK and activate BMP signals at the final stage of cultivation (days 30−34).

### Adult stem cells (ASCs) and pluripotent stem cells (PSCs) allow to establish the gastric organoids

Gastric organoids have been generated from stomach tissue-derived ASCs and PSCs. The main differences between these two types are the presence of mesenchymal cells in the cultivation of PSC-derived organoids. PSC-derived organoids are needed a stepwise protocol that differentiates PSCs into the targets identically; in contrast, ASC-derived gastric organoids require only a simple growth factor-enriched medium. Thus, the length of the cultivation needed to establish the organoids from PSCs is 1 or 2 months, whereas that of ASC-derived organoids is 1 or 2 weeks.

Mouse gastric organoids derived from ASCs were established from antrum glands containing LGR5^+^ stem cells. The procedure used in this experiment was the same as that employed for the intestinal organoid culture system, with addition of FGF10 and gastrin [37]. The expression of pepsinogen C as a marker of chief cells and MUC6 as a marker of mucus neck cells was also identified. The decreased concentration of WNT resulted in generating the differentiated lineages of endocrine cells and mucous pit cells, but not of parietal cells. Similar conditions for cultivation were used for producing murine corpus organoids from TROY^+^ stem cells [38]. These organoids expressed the markers of chief cells and mucus neck cells. In the absence of FGF10, NOGGIN, and WNT, differentiated pit cells could be found, whereas endocrine or parietal cells were not detected.

Human antral organoids can be generated using the mouse procedure described previously [39]. The generation of human corpus organoids with successful long-term proliferation required the inhibition of TGFβ signaling by A83-01 (an activin receptor-like kinase ALK5 inhibitor) [40].

Gastric organoids containing both epithelial and mesenchymal cells can be generated by the differentiation of PSCs. McCracken et al. reported the differentiation procedure from human PSCs to gastric organoids firstly [6, 41]. Addition of activin A and *BMP4* caused human PSCs to differentiate into endoderm. Activin A stimulated Nodal, which is critical for formation of foregut. Posterior foregut formation was succeeded by addition of FGF4 and WNT or CHIR99021, which is a glycogen synthase kinase 3 (GSK3) beta inhibitor that activates WNT signaling. Noggin was additionally required to inhibit the BMP signal and generate the stomach-derived foregut. The embedding of these cells into the extracellular matrix helped produce 3-D foregut spheroids. Differentiation of antrum was performed by incubation of retinoic acid (RA) and EGF. The complete differentiation process required approximately 34 days to produce neutral organoids containing enteroendocrine cells, mucus neck, and pits [6].

To develop the foregut into corpus organoids, supplementation with CGIR99021, EGF, and FGF10 was also required. To produce parietal cells, the medium was subsequently added by BMP4 and the MEK inhibitor PD0325901 [41]. Differentiated corpus organoids comprised a variety of key cells included chief, endocrine, mucus neck, and parietal cells [44]. Noguchi et al. generated organoids from murine PSCs using a stepwise differentiation protocol [42]. The PSC-derived embryoid bodies were treated with Noggin, sonic hedgehog (SHH), and the WNT antagonist Dickkoph 1 (DKK1). The mutual control of SHH activation and WNT inhibition allowed the formation of tube-like structures and spheroids that mimicked the structure of the early stomach. The embedding of spheroids resembled the early stomach-like matrix by adding FGF10, NOGGIN, WNT, and R-SPONDIN and allowed corpus gland formation after approximately 60 days.

A co-culture of ASC-derived organoids with mesenchymal cells overcomes the limitation of ASC-derived organoids inherent to their epithelial composition [43]. The addition of mesenchymal niche cells led to the production of all type cells of the stomach epithelium included parietal cells, although they were found only for a limited time.

Normal gastric organoids also represent a good model system for translational studies. Organoids can be used to recapitulate development of organs in vivo and represent a good model system to investigate *Helicobacter pylori* infection [44]. In addition, organoid systems have been successfully used for disease modeling by the recent gene editing techniques, such as CRISPR/Cas9 [45] and CRISPR/HAT [46]. Thus, we should focus on the generation of human biobanks for basic and clinical use of patient-derived gastric cancer organoids.

### Patient-derived gastric cancer organoids

Five independent researchers reported the generation of gastric cancer organoids (Table 2). Seidlitz et al. made a biobank composed of human gastric cancer organoids (20 organoids) with a molecular characterization of four additional organoid lines [47]. Vlachogiannis et al. generated a patient-specific biobank comprising different gastrointestinal cancers, including four gastric cancer organoids [48]. Nanki et al. produced a biobank of 37 gastric cancer organoids [49]. They clarified the effect of different niches on the support of individual cancers. Recently, a largest biobank was reported by Yan et al. [50], consisting of 46 organoid lines. Of interest, this report compared the organoids derived from multiple biopsies of the same patient, thus allowing the characterization of subclones within the primary cancer. Ukai et al. generated and characterized 10 gastric cancer organoids from surgically resected specimens, four of which were 5-FU-resistant organoids [51]. They identified the role of *KHDRBS3* as a gene involved in drug resistance in gastric cancer organoids. Various protocols using different enzymatic digestions have been used, such as dispase II and collagenase XI [50], EDTA and TrypLE [48], Liberase TH and TrypLE Express [49], collagenase and hyaluronidase [50], or Partec CellTrics (Sysmex, Hyogo, Japan; [51]). Growth medium composition also varied among the protocols (Table 2). To avoid cell contamination in the organoid preparations, Nanki et al. established a new method to enrich the cancer organoids by inhibiting various signals, i.e., RAS-phosphoinositide 3-kinase (PI3K), RHO, TGFβ, TP53 which are not tolerated by nonmutated normal organoids [49]. This protocol resulted in an increase in the efficiency of cancer organoid generation, from 55 to 75%. In contrast, Yan et al. enriched cancer organoids by microscopical selection, and by treatment with nutlin3a in the case of TP53 mutation (Table 3) [50].

**Table 3:** Patient-specific cancer organoids biobank characteristics

	Vlachogiannis et al. [48]	Seidlitz et al. [47]	Yan et al. [50]	Nanki et al. [49]	Ukai et al. [51]
Tissue origin	Ultrasound CT-guided biopsy	Surgical resection	Surgical resection	Surgical resection Endoscopic biopsy Ascites punctures	Surgical resection Endoscopic biopsy
Organoid library					
Cancer	4	20	46	37	20
Normal	**−**	3	17	6	10
Patients with clinical trails	**+**	**−**	**-**	**-**	**-**
Tissue digestion	TrypLE (2 x) EDTA (1 mM)	Dispase II (1 mg/ml) Collagenase (0.6 mg/ml)	Collagenase XI ( 0.1 μg/μl) Hyaluronidase (20 μg/μl)	Libease H TrypLE Express	Partec CellTrics (Sysmex, Hyogo, Japan) EDTA (1 mM)
**Medium composition**					
WNT3A	100 ng/ml	50%	50%	25%	25%
R-spondin	500 ng/ml	10%	10%	1 μg/μl	1 μg/μl
Noggin	100 ng/ml	10%	10%	100 ng/ml	100 ng/ml
B27	1x	1x	1x	1x	1x
N2	1x	1x	−	−	−
Nicotinamide	4 mM	10 mM	−	−	−
NAc-Cys	−	1 mM	1 mM	1 mM	1 mM
hFGF10	10 ng/ml	200 ng/ml	200 ng/ml	50 ng/ml	50 ng/ml
hFGF10-basic	10 ng/ml	−	−	−	−
mEGF	50 ng/ml	50 ng/ml	50 ng/ml	50 ng/ml	50 ng/ml
Gastrin	10 nM	1 nM	1 nM	10 nM	10 nM
A83-01	0.5 μM	2 μM	2 μM	500 nM	500 nM
Y-27632	10 μM	10 μM (only for initial stage)	10 μM	−	10 μM (only for initial stage)
Prostaglandin E2	1 μM	−	−	−	−
SB202190	5 μM	−	−	−	−
**Selection of cancer organoids from prevent normal organoid-overgrowth**	**−**	**−**	**+**	**+**	**+**
**Selection via**			1. Microscopically organoid picking 2. Nutlin3a (10 μM)	1. Plus Nutlin3a (3 μM) minus Y-27632 2. Minus A83-01 plus TGFβ (10 ng/ml) 3. Minus EGF and FGF18	1. Microscopically organoid picking 2. Drug resistant GCOs clones with selected by 5-FU, puromycin, and G418

Recent five independent groups reported the human gastric organoid preparation [see ref. 46‒50] and compared the culture conditions

+ : Yes, done -: none or not specified

### Morphological features

Seidlitz et al. reported the generation of cystic organoids with a multilayered wall that exhibited a growth pattern with noncoherent grape-like compact cell cluster without lumen [47]. Nanki et al. and Yan et al. [49, 50] grouped them into three subtypes, as shown in the Lauren classification [52]: (1) a solid subtype derived from diffused gastric cancers with amorphous solid configurations and a dis-cohesive growth pattern, (2) a glandular subtype derived from intestinal cancers with a single lumen, and (3) a mixed subtype [49–52].

A study between phenotype and genotype was reported by Nanki et al., who knocked out *CDH1* using CRISPR/Cas9, which allowed the phenotypical alteration of organoids from normal cystic structures to slide structures with migration activity, resembling patient-specific organoids with a *CDH1* mutation [49]. This trial should be continued to address the relationship between molecular mechanisms and the morphological and histological alterations of the HGOs.

### Molecular characterization

The Cancer Genome Atlas Research Network [53] demonstrated the existence of the MSI, GS, CIN, and EBV subtypes among patient-specific gastric cancers. In addition, a 96% overlap in the mutational spectrum between organoids and parental organs was identified [47, 48]. Yan et al. reported varying degrees of heterogeneity in gastric cancer by comparing patient-specific gastric cancer organoids from primary tumors and lymph node metastases [50]. A patient-specific gastric cancer organoid library of different tumors from an individual patient generates a new tool that can be used to investigate the consequent outputs of intra-tumoral heterogeneity. Nanki et al. investigated the correlation between phenotype and genotype by focusing on the niche-derived factor dependences and genetic alterations [49]. Ki-ras2 Kirsten rat sarcoma viral oncogene homolog (*KRAS*) mutation and RTK amplification, similar to that observed for the erb-b2 receptor tyrosine kinase 2 (*ERBB2*) or *ERBB3*, led to the acquisition of independency from both EGF and FGF. The high expression of epiregulin (EREG), a ligand of EGFR, mediated the independence from EGF and FGF, suggesting an EREG-dependent autocrine loop.

The inhibition of TGFβ and BMP is important for the growth of the organoids. Treatment of organoids with mutations in the TGFβ-receptor 2 (*TGFBR2*) and the SMAD family member 4 (*SMAD4*) genes with TGFβ and BMP4 did not affect their proliferation rate. Some cancer organoids without any mutations in the genes mentioned above were resistant to stimulation with TGFβ and BMP4, thus prompting the search for additional nongenetic mechanisms that allow tolerance toward TGFβ and BMP among patient-specific gastric cancer organoids [45]. Thus, this mechanism should be explored further to identify target genes.

WNT and R-spondin are also critical for the successful proliferation of normal gastric organoids. Thus, the gastric cancer organoids acquire WNT independently during tumor progression, e.g., by mutation of the adenomatous polyposis coli (APC) gene. Another mechanism consisting in the activation of WNT ligand production allows the autocrine stimulation of the tumor using an inhibitor of WNT ligand production [54]. R-spondin binds to LGR4/5 and stabilizes the frizzled and LRP as WNT receptors. In the absence of R-SPONDIN, both RNF43 and ZNRF3 are capable of ubiquitinating WNT receptors [53]. *RNF43* mutations are detected in only 5% of microsatellite-stable tumors, but exhibit a high mutation ratio in MSI-subtype patients (55%) [55]. Of interest, several stomach and intestinal cancer organoids carrying the single *RNF43* mutation remained R-SPONDIN dependent [53]. Subsequent genetic studies revealed that double mutations, homologous deletions, and downregulation of the RNF43 mRNA and the corresponding homolog of ZNRF3 caused R-SPONDIN independently. Interestingly, this was also observed for a RNF43D300Y single mutation. Thus, Wnt receptor regulators and TGFβ−BMP signaling are critical for the growth of cancer organoids.

### Several critical growth factors are important for the growth and proliferation of gastric organoids

**Fig. 4:**
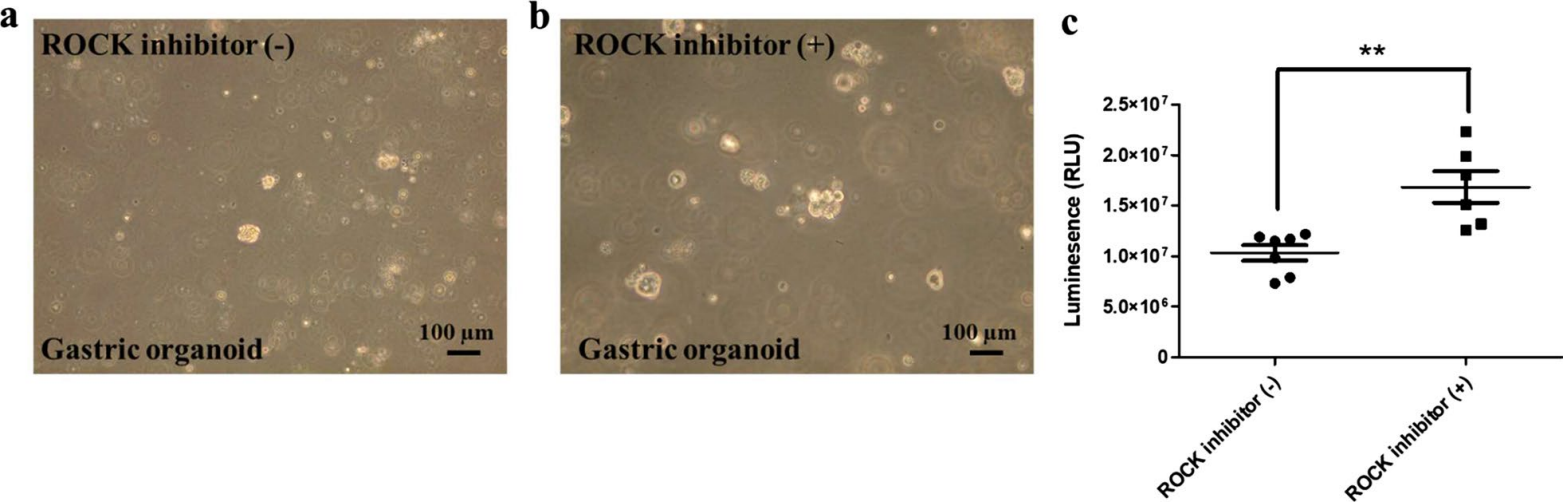
Effect of ROCK inhibitor in gastric organoid culture. The rock inhibitor supplied in culture medium maintained the growth and morphologies of the organoids well and helped to avoid the culture-damages caused by the culture-environments. (**a**) The cultivation in the absence of the ROCK inhibitor (left lane) caused the growth of the organoids slowly. (**b**) Addition of ROCK inhibitor (10 µM; right panel) resulted in the growth of the organoids 1.5–2.0-fold faster compared with the control organoids (left panel). Human adenocarcinoma of stomach organoid lines, HCM-BROD-0208-C16 cancer model primary adenocarcinoma of stomach was purchased from American Type Culture Collection (PDM-146™; ATCC, VA, USA) and cultured as its recommended protocol [5] (bright view, scale bar = 100 μm). (**c**) The stomach organoids were generated by seeding in 96-well plates and grown 2 days ROCK inhibitor (n = 6): Th organoids were incubated with CellTiter-Glo®3D reagent (G9681, Promega, Madison, WL, USA) prior to culture medium addition, and results were acquired to assay (** *P* < 0.01).

**Fig. 5:**
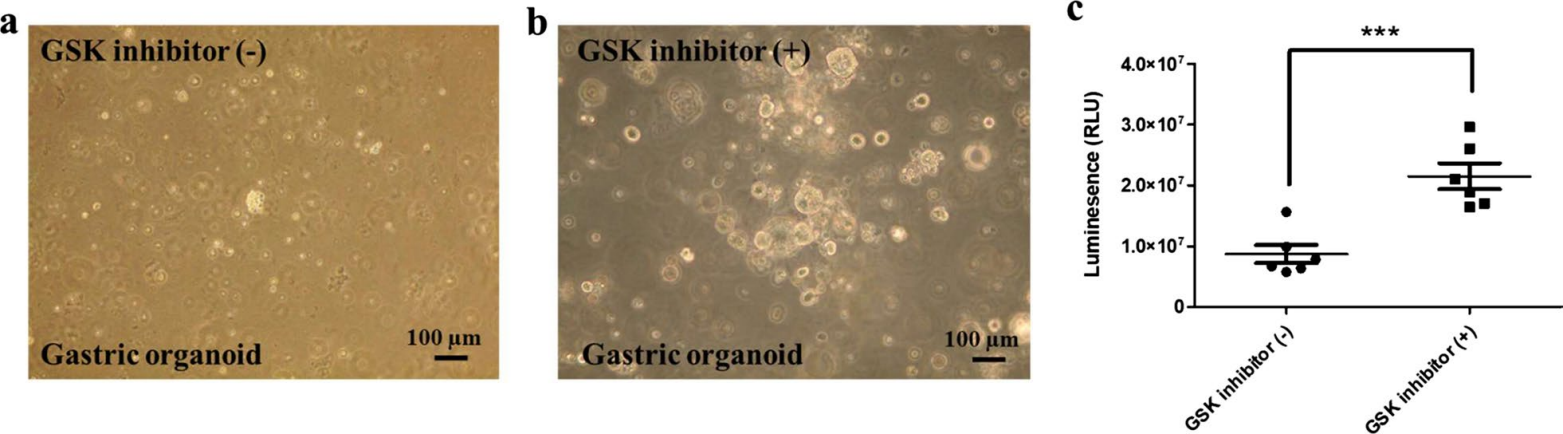
Effect of GSK inhibitor in gastric organoid culture. The gastric organoids were fragile and damaged easily during the passaging process. (**a**) Organoids without GSK inhibitor resulted in apoptotic deaths and damaged the formation of the 3-D structure. (**b**) The addition of GSK inhibitor (2 µM) supplied in every 2–3 days dramatically reduced and protected the cells-damage and stimulated the cell growth 2-threefold faster than in the control culture of the organoids. Human adenocarcinoma of stomach organoid lines, HCM-BROD-0208-C16 cancer model primary adenocarcinoma of stomach was purchased from American Type Culture Collection (PDM-146™; ATCC, USA) and cultured as its recommended protocol [5] (bright view images, scale bar = 100 μm). (**c**) The stomach organoids were generated by seeding in 96-well plates and grown 2 days GSK inhibitor (n = 6): Th organoids were incubated with CellTiter-Glo®3D reagent (G9681, Promega, Madison, WL, USA) prior to culture medium addition, and results were acquired to assay (*** *P* < 0.001)

**Fig. 6:**
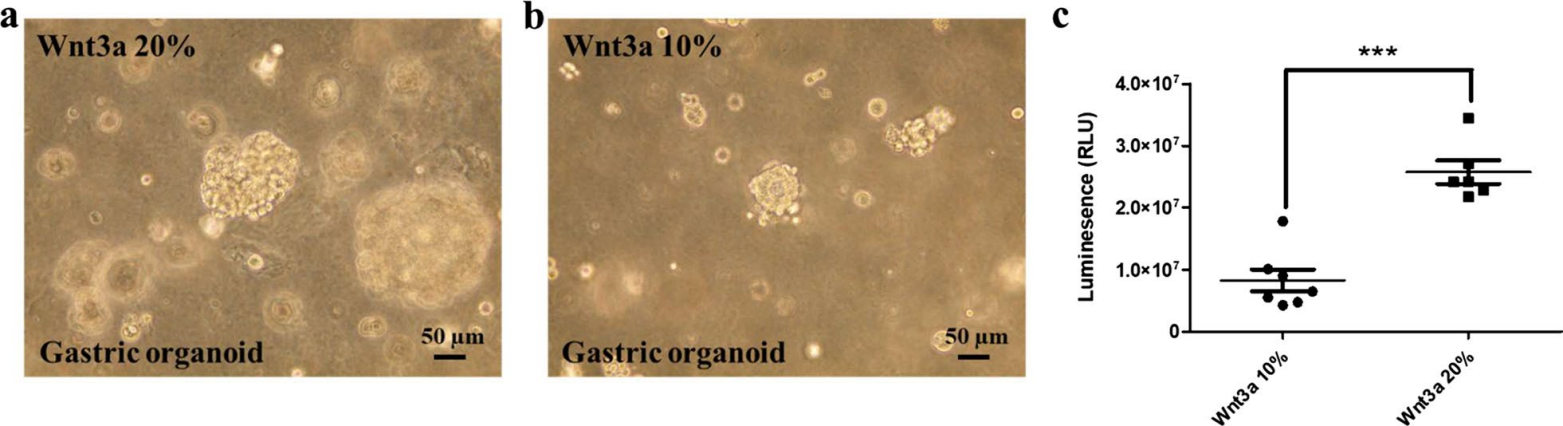
Effect of niche factors in gastric organoid culture. The concentration of R-SPONDIN 1 in the gastric cancer organoids culture was 10%, and the concentration of Wnt3a in the cancer organoids was 20% (panel **a**) and 10% (panel **b**). Human adenocarcinoma of stomach organoid lines, HCM-BROD-0208-C16 cancer model primary adenocarcinoma of stomach was purchased from American Type Culture Collection (PDM-146™; ATCC, USA) and cultured as its recommended protocol [5] (bright view images scale bar = 50 μm). (**c**) The stomach organoids were generated by seeding in 96-well plates and grown 2 days at different percentages of Wnt (n = 6). Th organoids were incubated with CellTiter-Glo®3D reagent (G9681, Promega, Madison, WL,USA) prior to culture medium addition, and results were acquired to assay (*** *P* < 0.001),

To clarify the fidelity of human organoid models, we have noted several key issues for the cultivation and differentiation of normal and cancerous organoids (Unpublished data; Ref. [5]). (1) Effect of ROCK inhibition: RHO kinase inhibitor was added to inhibit the anoikis and apoptosis observed previously in purified colonic epithelial cells. This is absolutely required for organoid growth from the establishment stage (Fig. 4). (2) Effect of GSK inhibition: treatment with CHIR-99021 (a GSK inhibitor) and activated β-catenin-mediated transcription and induced phenotypic alterations in organoids. Of note, CHIR treatment with HDAC inactivation via the administration of valproic acid represented an effective method for the enrichment of LGR5^+^ cells in intestinal organoids. Thus, we used this protocol for 3 days and then removed it, which led to good growth of the organoids (Fig. 5). (3) Dose of niche factors: R-SPONDIN 1 concentration was similar in normal and cancer organoids (10%), whereas the concentration of WNT3a 20% in cancer cases was better than 10% to obtain the good proliferation (Fig. 6). We are developing precise culture conditions for gastric organoids in vitro, to improve the accuracy of the generation of organoid models for future therapeutic and medical applications.

## HGO application

### Tissue engineering

The use of organoids for clinical use as replacement tissues faces significant challenges in a near future. The stomach in humans is about 10 cm × 30 cm, while stomach organoids are in diameter 2−3 mm. Thus, the requirement for new engineering of the tissue-growth formation for scaling-up is required because of the size gap between the tissues and the organoids of the stomach. One possible route should be in vivo growth, as described for intestinal organoids, which become larger than the original size [56]. The next problem lies in the complexity of the stomach. For example, HGOs contain an epithelium and mesenchymal layer and are deficient in ENS, which is crucial for stomach functions. This problem has been solved as reported in the case of human intestinal organoids. Previously, the successful incorporation of ENS within the mesenchyme layer of the human intestinal organoids was reported [56]. In the future, this will be carried out using HGOs. Another trial incorporated a vascular function into human lung organoids using a combination of endothelial cells and the immune system to generate human colonic and liver organoids, as reported [57]. Thus, the combination between different germ layers and different cell types within hPSC-derived gastrointestinal organoids might help understand the role of each layer and cell type in the creation of the tissue models.

### *Helicobacter* (H.) *pylori* infection

Gastroenteritis, gastritis, gastroparesis, peptic ulcer disease, and stomach cancer are well-known diseases of the stomach, with the latter three being associated with the *Bacillus* bacterium *H. pylori* and gastritis [58]. These diseases are featured by inflammation and erosion, or irritation of the lining of the stomach. Patients with *H. pylori* infection have a 10−20% frequency of developing ulcers and gastric cancers at 1−2% risk. Among patients with ulcers, 95% of patients with duodenal ulcers and 80% of patients with gastric ulcers are positive for *H. pylori* infection [59]. Ulcers appears to arise in the corpus–antrum transition area, as well as in the duodenum to study *H. pylori* pathogenesis [60].

HGOs derived from hPSCs provide a different model to investigate the stomach development. The signaling and interaction of the niches are studied by HGO-based platforms and might ideally be adapted to characterize the interactions of these various cell types during developing the human stomach. Finkbeiner et al. [61] reported the use of hSC-derived intestinal organoids to model a viral infection that supported the replication of rotavirus and the production of infection of both epithelial and mesenchymal components of the intestinal organoids, which was a promising platform not only for the study of the virus, but also other viral-related organisms. Forbester et al. [62] reported the injection of *Salmonella enterica* serovar *typhimurium* into the lumen of hPSC-derived intestinal organoids and demonstrated that the bacteria can attack the intestinal epithelium, form intracellular *Salmonella*-containing vacuoles, and induce the production of cytokines. These vacuoles required oxygen consumption. Leslie et al. [63] also reported the merits of organoid-based infection. Thus, this platform could be useful for the study of improved treatments via drug screenings related to pathogens, for which there are no efficient therapeutic agents.

### Drug discovery and testing

HGOs are also a new alternative tool to examine the toxicity of drugs and the in vitro screening of drugs and small molecules. For example, some drugs that block acid production are useful to inhibit acid secretion in fundic HGOs. It suggests that HGOs would be an ideal tool for searching for novel drugs and small molecules that regulate acid production [64]. This technology might provide a high-throughput method of investigating the efficacy and toxicity of drugs. Thus, technological improvements and challenges are key points in the development of HGO technologies for future therapeutic screening.

### Limitations of organoids

There are several limitations to the present organoid technology. (1) Growth factors are, in some cases, generated by cell lines expressing exogenous genes that encode them, rather than the common commercially available growth factors. This will reduce the cost of maintenance but increase the experimental variation produced by the niche factors, which is a tedious procedure. (2) Another limitation is the practical use of Matrigel or other animal-derived devices to enable cells to assemble into 3-D organoids. As the composition of these materials is not well defined, unforeseen problems might occur. Moreover, the removal of the Matrigel is critical for subsequent analysis, including extraction of DNA and RNA, CRISPR-Cas9 editing, or even cryopreservation. Gjorevski et al. [65] reported that a benefit of the high matrix stiffness to expand iPSCs through a YAP-mediated mechanism, while a soft matrix to increase the efficacy of laminin-based adhesion was required for iPSC differentiation and organoid generation. Such well-defined tools with minimal environment could accommodate the tissue dynamics that occur during the developmental processes. Another approach is the hydrogel-based platform that promoted the aggregation of progenitor cells into pancreatic organoids that maintained an islet morphology and function which were featured by enhanced expression of the PDX-1 and NKK6 [66]. (3) One more limitation is the lack of studies on immune interaction with gastric organoids. However, new technology to investigate the effect of immunoregulation on epithelial organoids was recently developed. Moreover, the technology employing fusion of organoids and coculture of multiple organoids has been developed recently [67]. The coculture of organoids with immune cells [68], ENS cells [56], and luminal factors such as bacterial cocultures [44] are important to study the microenvironmental regulation of CSCs. Many challenges should be remained in the long-term culture, and the preservation of immune cells could be stocked, and then, they are reconstituted. Supplements such as IL-2, antibodies against CD3 or CD28 might improve the long-term maintenance of immune cells, but we need the novel recapitulation of antitumor response by these combined organoids would be certainly necessary for robust validation [69, 70].

## Conclusion

HGOs are used to be a promising tool because they are managed to recapitulate the exact stomach in vivo and allow unprecedented investigations of human development. Their translational applications will promote the diagnosis of patient pathologies and the development of screening techniques for pharmacological drugs in the future. Patient-specific HGOs are also precious tools as we encounter the era of personalized medicine. Large human organoid biobanks generated from diverse cancer tissues have been established and characterized in detail. A recent study performed by Kawasaki et al. [71] reported the establishment of a gastro-entero-pancreatic (GEP) neuroendocrine neoplasm (NEN) organoid biobank characterized by NKX2-5 expression and exhibiting WNT- and EGF-independent growth. These studies are also useful to address the de novo modeling of GEP-NEN through the genetic engineering of normal and tumor gastric organoids. Human organoid libraries have merits to investigate cancer characteristics and novel treatments in patients. In near future, patient-derived organoids from normal tissues and cancer tissues can provide a useful bridge between molecular genetics based on genome wide research and clinical translation research.

## Data Availability

Please contact author for data requests.

## Abbreviations

ADCs: Antibody–drug conjugates
AMPK: Adenine monophosphate-activated protein kinase
APC: Adenomatous polyposis coli
ARIDIA: AT-rich interactive domain-containing protein 1A
ASCs: Adult stem cells
BMP: Bone morphogenic protein
CAFs: Cancer-associated fibroblasts
CSCs: Cancer stem cells
3-D: Three-dimensional
DKK1: WNT antagonist Dickkoph 1
EC: Enterochromaffin
ECMs: Extracellular matrices
EGL: Enterochromaffin-like
ERBB2: Erb-B2 receptor tyrosine kinase 2
EREG: Epiregulin
ESCs: Embryonic stem cells
FDA: Food drug administration
GAC: Gastric adenocarcinoma (GAC)
GEP: Gastroenteropancreatic
GSK3: Glycogen synthetase kinase 3
HGC: Human gastric cancer
HGOs: Human gastric organoids
IL6: Interferon 6
iPCCs: Induced pluripotent cancer cells
iPCSCs: Induced pluripotent cancer stem cells
iPSCs: Induced pluripotent stem cells
KRAS: Kirsten rat sarcoma viral oncogene homolog
MSCs: Mesenchymal stem cells
NEN: Neuroendocrine neoplasm
PcG: Polycomb repressive group
PSCs: Pluripotent stem cells
PTEN: Phosphatase and tensin homolog protein
RA: Retinoic acid
Shh: Sonic hedgehog
TGFβ: Transforming growth factor-β
TGFBR2: TGFβ-receptor 2
TrxG: Trithorax group

## Declarations

### Declaration of use of any animal and human data or tissues

This study was approved by Kaohsiung Medical University Hospital Ethical Committee (Approval numbers: E(I)-20170111 and E(II)-20170104). We used the human HCM-BROD-0208-C16 cancer model, and primary adenocarcinoma of stomach was purchased from American Type Culture Collection (PDM-146™; ATCC, VA, USA). No direct interaction with the study participants occurred, and no written informed consent for the use of the diagnostic sample for research was needed.
